# Tumor classification ranking from microarray data

**DOI:** 10.1186/1471-2164-9-S2-S21

**Published:** 2008-09-16

**Authors:** Rattikorn Hewett, Phongphun Kijsanayothin

**Affiliations:** 1 Department of Computer Science, Texas Tech University, Abilene, TX, 79601, USA

## Abstract

**Background:**

Gene expression profiles based on microarray data are recognized as potential diagnostic indices of cancer. Molecular tumor classifications resulted from these data and learning algorithms have advanced our understanding of genetic changes associated with cancer etiology and development. However, classifications are not always perfect and in such cases the *classification rankings *(likelihoods of correct class predictions) can be useful for directing further research (e.g., by deriving inferences about predictive indicators or prioritizing future experiments). Classification ranking is a challenging problem, particularly for microarray data, where there is a huge number of possible regulated genes with no known rating function. This study investigates the possibility of making tumor classification more informative by using a method for classification ranking that requires no additional ranking analysis and maintains relatively good classification accuracy.

**Results:**

Microarray data of 11 different types and subtypes of cancer were analyzed using MDR (Multi-Dimensional Ranker), a recently developed boosting-based ranking algorithm. The number of predictor genes in all of the resulting classification models was at most nine, a huge reduction from the more than 12 thousands genes in the majority of the expression samples. Compared to several other learning algorithms, MDR gives the greatest AUC (area under the ROC curve) for the classifications of prostate cancer, acute lymphoblastic leukemia (ALL) and four ALL subtypes: BCR-ABL, E2A-PBX1, MALL and TALL. SVM (Support Vector Machine) gives the highest AUC for the classifications of lung, lymphoma, and breast cancers, and two ALL subtypes: Hyperdiploid > 50 and TEL-AML1. MDR gives highly competitive results, producing the highest average AUC, 91.01%, and an average overall accuracy of 90.01% for cancer expression analysis.

**Conclusion:**

Using the classification rankings from MDR is a simple technique for obtaining effective and informative tumor classifications from cancer gene expression data. Further interpretation of the results obtained from MDR is required. MDR can also be used directly as a simple feature selection mechanism to identify genes relevant to tumor classification. MDR may be applicable to many other classification problems for microarray data.

## Background

Numerous studies have shown that cancer involve accumulated genetic aberrations in the cell. Advances in DNA microarray technology have revolutionized cancer research by enabling, within a given cell population, the simultaneous monitoring of the transcription and complex changes in the expression of thousands of genes during cancer development. This makes rapid genetic analysis for genome-wide cancer studies feasible. Researchers can quickly compare gene expressions between normal and malignant cells, and explore the genetic changes associated with cancer etiology and development. Microarray analysis offers promising avenues to the discovery of both new biomarkers for cancer diagnosis and prognosis and new treatments. Microarray data are being used to categorize tumors on the basis of their molecular profiles, to identify subtypes of tumors, to predict patients' responses to treatment and risk of relapse, and to explore the biological properties of tumors [1-7].

Recent cancer research has applied a variety of machine learning algorithms for tumor prediction by associating expression patterns with clinical outcomes for patients with tumors in various stages [3,4,8,9]. Due to the distinctive huge dimensionality of the data, the majority of research has focused on building accurate classification models from reduced sets of features. The analysis aims to gain understanding of the differences between normal and malignant cells and to identify genes that are differentially regulated during cancer development. While this is useful, when classification models are not 100% accurate, the likelihoods of correctness for the class predictions (i.e., *classification ranking*) can be useful for further research (e.g., deriving inferences for predictor genes and prioritizing experiments). For example, some of the genetic abnormalities in malignant cells may be the most important contributing factors for cancer. Classification ranking is a challenging problem, particularly in microarray data, which has a huge number of factors whose relative importance is largely unknown. Most machine learners focus on classification and do not explicitly assess the likelihood of correctness for their class predictions, unless additional analysis is performed.

This paper describes a simple microarray data analysis technique for tumor classification ranking. In particular, we apply MDR, our recently developed Multi-Dimensional Ranking algorithm, for analyzing gene expression in various types of cancers including leukemia, lung, prostate, lymphoma, and breast cancers. These data have been used in previous cancer research studies [1,3,5-8,10]. They are publicly available and can be obtained from the Kent Ridge Biomedical Data Set Repository [11].

## Results

We analyze microarray data for 11 types and subtypes of tumors using MDR. The two Leukemia expression data sets are concerned with classification of acute lymphoblastic leukemia (ALL). Golub et al. [8] used the ALL-AML Leukemia expressions to help discover a single diagnostic test to differentiate between two *types *of human acute leukemia: acute myeloid leukemia (AML) and ALL. The ALL-subtype expression data were used by Yeoh et al. [7] to identify six known prognostically important leukemia *subtypes *of ALL from pediatric ALL patients. These ALL subtypes include: BCR-ABL, E2A-PBX1, Hyperdiploid > 50 chromosomes, MALL, T-ALL, and TEL-AML1. Because different leukemia types and subtypes respond to chemotherapy differently, the ability to determine the classification of an ALL subtype for a new leukemia tissue sample can be valuable for cancer treatment. MDR has been designed for binary classification ranking. When dealing with multiple classes, we employ the "one against many" strategy (i.e., for each class, perform a binary classification between that class and all the other classes). As recommended in a recent study by Li and Liu [9], we divide the ALL-subtype expression data into six cases, each of which focuses on classification of a particular ALL subtype against all the other subtypes. The ALL classification model (or classifier) obtained from MDR contains two predictor genes, whereas the number of predictor genes in the classifiers for the six ALL subtypes ranges from one to nine. This is a huge reduction from the original number of genes, over 12,000, in the ALL-AML and ALL subtype expression data.

The lung cancer data are analyzed to distinguish between malignant pleural mesothelioma (MPM) and adenocarcinoma (ADCA) [3], whereas the prostate and breast profile expression data are analyzed for tumor diagnosis and prognosis (e.g., "relapse" in patients who developed distance metastates within five years), respectively [5,6]. The lymphoma microarray data include gene expressions of diffuse large B-cell lymphoma, a subtype of non-Hodgkin's lymphoma [1]. To indicate different stages of B-cell malignancies, gene expression patterns studied are of two types: the germinal centre B-like type and the activated B-like type. Patients with the germinal centre B-like type have had better survival rates. The classification models obtained by MDR from the lung, prostate, lymphoma and breast expression data contain five, six, three and eight predictor genes, respectively. The predictor genes in each of these MDR classification models, derived from the corresponding expression data sets, are shown in Table 1. While constructing the classification model, MDR reduces the number of data dimensions to a smaller number, selecting features relevant for classification ranking in the model. Unlike the machine learners that require an additional step for feature selection, MDR does not do feature selection as a separate step. MDR could be viewed as an alternative, simple method for a boosting-based feature selection technique. However, proper verification of MDR's effectiveness as a method for feature selection would require research beyond the scope of this paper.

**Table 1:** Features in the classification models obtained by MDR.

Name	Features
ALL-AML Leukemia	attribute1834, attribute6855
Lung cancer	37954_at, 33328_at, 1500_at, 34320_at, 37716_at
Prostate cancer	37639_at, 34163_g_at, 38406_f_at, 1776_at, 33784_at, 32057_at
Lymphoma	GENE3328X, GENE3512X, GENE3261X
Breast cancer	AL080059, AF035278, AB014543, Contig16531_RC, Contig64861_RC, NM_004469, Contig34634_RC, Contig15044_RC
BCR-ABL	1636_g_at, 36591_at, 37602_at, 40698_at
E2A-PBX1	32063_at
Hyp	31308_at, 38461_at, 37543_at, 1916_s_at, 36620_at, 39721_at, 36517_at,
	38402_at
MALL	33412_at, 31397_at, 34306_at, 31318_at, 40506_s_at, 31329_at,
	38413_at, 31324_at, 36777_at
TALL	38319_at
TEL-AML1	36985_at, 31572_at, 31492_at, 36239_at, 32645_at, 31691_g_at

**Figure 1:**
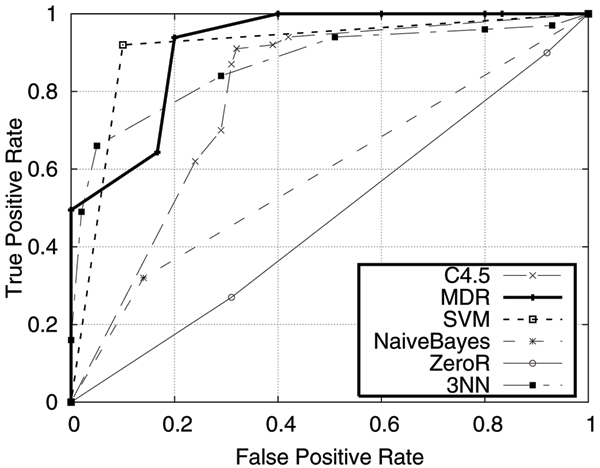
ROC curves on prostate cancer expressions

The performance of MDR is compared to that of other high performance machine learners in Weka [12]. Figure 1 shows a comparison of ROC curves obtained from MDR and five other learners (ZeroR, C4.5, Bayes, 3NN, and SVM) for the prostate cancer data set. The ZeroR learner predicts values based on the majority of the class distribution and is commonly used as a baseline measure of performance. As expected, ZeroR shows performance equivalent to random guessing, giving an ROC curve close to a diagonal line. Therefore, it has no predictive power. The ROC curves obtained from C4.5 and Bayes show better performance than ZeroR. The top three performers appear to be MDR, 3NN and SVM. Both MDR and SVM dominate the others but we cannot draw a firm conclusion on comparative performance by looking at the ROC curve alone. While the ROC curve is effective for ranking quality, it is not always conclusive. Thus, our analysis employs alternative standard performance measures (as described in the Method section), including *recall *(true positive rate, sensitivity, hit rate), *FPR *(false positive rate, false alarm rate, type I error), *FNR *(false negative rate, type II error), *ACC *(accuracy), *AUC *(area under ROC curve) and precision. The results of these measures (shown as average percentages over 10-fold cross validation) obtained from six learners are given in Table 2, for the classification of five types of cancer, and Table 3, for the classification of six subtypes of acute lymphoblastic leukemia (ALL).

**Table 2:** Classification results on five cancer types.

ALL-AML Leukemia						
Learner	Recall	FPR	FNR	ACC	AUC	Precision

C4.5	85.11	32.00	14.89	79.17	72.00	83.33
Bayes	100.00	4.00	0.00	98.61	97.90	97.92
3NN	95.74	40.00	4.26	83.33	87.50	81.82
SVM	100.00	4.00	0.00	98.61	98.00	97.92
ZeroR	100.00	100.00	0.00	65.28	42.60	65.28
MDR	97.87	4.00	2.13	97.22	99.02	97.87

Lung cancer						

Learner	Recall	FPR	FNR	ACC	AUC	Precision

C4.5	87.10	3.33	12.90	95.03	93.00	84.38
Bayes	96.77	1.33	3.23	98.34	97.70	93.75
3NN	64.52	0.00	35.48	93.92	96.50	100.00
SVM	96.77	0.00	3.23	99.45	98.00	100.00
ZeroR	0.00	0.00	100.00	82.87	48.50	nan
MDR	90.32	2.00	9.68	96.69	97.61	90.32

Prostate cancer						

Learner	Recall	FPR	FNR	ACC	AUC	Precision

C4.5	87.01	30.51	12.99	79.41	79.00	78.82
Bayes	32.47	13.56	67.53	55.88	59.50	75.76
3NN	84.42	28.81	15.58	78.68	87.10	79.27
SVM	92.21	10.17	7.79	91.18	91.00	92.21
ZeroR	100.00	100.00	0.00	56.62	47.90	56.62
MDR	89.61	13.56	10.39	88.24	92.30	89.61

Lymphoma cancer						

Learner	Recall	FPR	FNR	ACC	AUC	Precision

C4.5	69.57	12.50	30.43	78.72	77.00	84.21
Bayes	100.00	4.17	0.00	97.87	97.90	95.83
3NN	60.87	8.33	39.13	76.60	81.30	87.50
SVM	100.00	4.17	0.00	97.87	97.90	95.83
ZeroR	0.00	0.00	100.00	51.06	40.80	nan
MDR	86.96	12.50	13.04	87.23	93.61	86.96

Breast cancer						

Learner	Recall	FPR	FNR	ACC	AUC	Precision

C4.5	52.17	27.45	47.83	62.89	66.00	63.16
Bayes	4.35	0.00	95.65	54.64	52.20	100.00
3NN	45.65	31.37	54.35	57.73	59.50	56.76
SVM	69.57	33.33	30.43	68.04	68.10	65.31
ZeroR	0.00	0.00	100.00	52.58	46.50	nan
MDR	60.87	35.29	39.13	62.89	63.65	60.87

**Table 3:** Classification results on six subtypes of acute lymphoblastic leukemia.

ALL-BCR-ABL						
Learner	Recall	FPR	FNR	ACC	AUC	Precision

C4.5	33.33	2.88	66.67	94.19	59.10	35.71
Bayes	0.00	0.32	100.00	95.11	49.80	0.00
3NN	13.33	0.00	86.67	96.02	75.10	100.00
SVM	26.67	0.00	73.33	96.64	63.30	100.00
ZeroR	0.00	0.00	100.00	95.41	41.40	nan
MDR	20.00	3.85	80.00	92.66	84.65	20.00

ALL-E2A-PBX1						

Learner	Recall	FPR	FNR	ACC	AUC	Precision

C4.5	100.00	0.00	0.00	100.00	100.00	100.00
Bayes	3.70	0.00	96.30	92.05	53.70	100.00
3NN	92.59	0.00	7.41	99.39	99.00	100.00
SVM	96.30	0.00	3.70	99.69	98.10	100.00
ZeroR	0.00	0.00	100.00	91.74	46.10	nan
MDR	100.00	0.00	0.00	100.00	100.00	100.00

ALL-HYP						

Learner	Recall	FPR	FNR	ACC	AUC	Precision

C4.5	65.63	6.08	34.38	88.38	79.50	72.41
Bayes	20.31	1.52	79.69	83.18	60.20	76.47
3NN	73.44	1.52	26.56	93.58	93.30	92.16
SVM	87.50	0.76	12.50	96.94	93.40	96.55
ZeroR	0.00	0.00	100.00	80.43	47.70	nan
MDR	37.50	9.13	62.50	80.43	85.44	50.00

ALL-MALL						

Learner	Recall	FPR	FNR	ACC	AUC	Precision

C4.5	80.00	1.95	20.00	96.94	89.00	72.73
Bayes	0.00	0.33	100.00	93.58	49.80	0.00
3NN	50.00	0.00	50.00	96.94	86.50	100.00
SVM	70.00	0.00	30.00	98.17	85.00	100.00
ZeroR	0.00	0.00	100.00	93.88	49.70	nan
MDR	45.00	3.58	55.00	93.27	88.90	45.00

ALL-T-ALL						

Learner	Recall	FPR	FNR	ACC	AUC	Precision

C4.5	100.00	0.35	0.00	99.69	99.80	97.73
Bayes	23.26	0.35	76.74	89.60	61.50	90.91
3NN	81.40	0.00	18.60	97.55	94.40	100.00
SVM	97.67	0.00	2.33	99.69	98.80	100.00
ZeroR	0.00	0.00	100.00	86.85	47.20	nan
MDR	100.00	0.00	0.00	100.00	100.00	100.00

ALL-TEL-AML1						

Learner	Recall	FPR	FNR	ACC	AUC	Precision

C4.5	87.34	2.42	12.66	95.11	92.50	92.00
Bayes	37.97	2.42	62.03	83.18	70.60	83.33
3NN	93.67	3.63	6.33	95.72	97.90	89.16
SVM	100.00	1.61	0.00	98.78	99.20	95.18
ZeroR	0.00	0.00	100.00	75.84	49.10	nan
MDR	82.28	5.65	17.72	91.44	91.50	82.28

Consider the results shown in Table 2. For prostate cancer, the AUCs obtained by all the learners are consistent with the observations from the ROC curves shown in Figure 1, but now we can make conclusive comparisons. Based on AUC, MDR, at 92.3%, and is slightly better than SVM, 91%. However, when considering all other measures, SVM generally performs better than MDR with differences of no more than 3.39%. AUC ranks the results differently than the other measures because it is a more suitable measure for ranking evaluation. Nevertheless, MDR outperforms 3NN by larger differences (9.56% in accuracy, 10.34% in precision, 15.25% in FPR (Type I error) and about 5% in AUC, recall and FNR (Type II error)). Both the accuracies and AUCs obtained from C4.5 and Bayes are much lower than those of MDR. For prostate cancer classification, MDR can rank well (with over 90% AUC) while its classification quality remains competitive with the other learners (close to 90% for accuracy, recall and precision).

For ALL-AML Leukemia, the top three performers are SVM, Bayes and MDR. All have the same FPR of 4%. SVM and Bayes have the highest recall, accuracy, precision and the lowest FNR. However, MDR has the best AUC of 99.02%, and the second best values for the other measures, with precision lower by only 0.04%, accuracy lower by about 1%, and differences in FNR and recall of about 2%. This is similar to the results obtained in prostate cancer. However, here SVM and Bayes give highly competitive results in all measures except AUC. On the other hand, MDR has reasonably high precision, accuracy and recall (97.87%, 97.22%, and 97.87%, respectively). MDR performs very well on the classification ranking of ALL. MDR's performance on lung cancer is similar to its performance on lymphoma cancer. For lung cancer, SVM, Bayes and MDR consistently performed better than the rest. The AUC and ACC obtained by MDR were 0.39% and 2.76% lower than those of the top performer, but it still has over 90% on almost all measures, with a low 2% FPR. Unfortunately, the 9.68% for FNR obtained by MDR is rather high, but still better than the 12.9% and 35.48% of C4.5 and 3NN, respectively. In the lymphoma cancer analysis, Bayes and SVM are top performers, followed by MDR. The AUC for MDR is 93.61%, which is 4.29% lower than that of Bayes and SVM. Compared to MDR, 3NN has 4.17% lower FPR (type I error) but 25.8% higher FNR (type II error), which is usually a more critical measure for tumor prediction. The ACC and AUC obtained by C4.5 and 3NN are not competitive to those obtained by MDR.

All the machine learners had poor results for the breast cancer data. Furthermore, the results obtained from different measures are not consistent. For example, Bayes has 100% precision but very low AUC and ACC of only about 50–55%, and an extremely high Type II error of about 95%. This suggests that the data are probably very skewed. The results obtained from SVM are the best with AUC, ACC and recall all close to 70%. However, its precision is only 65%, and it has very high type I & II errors of about 30%. MDR does not perform well. Nevertheless, the majority of its results rank second best among all the learners. We now discuss the results obtained in the ALL subtype classification as shown in Table 3. For the classification of subtypes E2A-PBX1 and T-ALL, MDR has perfect averages in all measures. For the subtype BCR-ABL, although MDR has the highest AUC, 88.48%, and high accuracy, 92.66%, (3.98% lower than the accuracy obtained by the best learner, SVM), it has low precision, low recall, and high FNR, about 80%. The results obtained by the other learners also show similar inconsistency across different measures. Thus, for BCR-ABL classification, all learners are likely to perform well but with high variances. For the rest of the ALL subtypes: Hyperdiploid > 50 chromosomes (HYP > 50), MALL and TEL-AML1, the results are similar in that the top three performers are SVM, 3NN, and C4.5 on almost all measures except AUC. However, MDR gives reasonably high accuracy, ranging from 80% to 93%, and AUC ranging from 85% to 91%. Since AUC is the area under the ROC curve, which is a plot between TPR and FPR, it may seem strange that a lower TPR and higher FPR can yield a higher AUC. However, this is possible when multiple points on the curve are considered (e.g., results obtained by MDR and C4.5 in HYP > 50). This is why AUC is one of the most widely used measures for evaluating probabilistic classification models [13].

To better understand MDR's performance for various types of cancer microarray data analyses, Table 4 focuses on comparisons of AUC in average percentages over 10-fold cross validations. For each type and subtype of tumor classification, the base line performance (ZeroR) gives an average AUC ranging from 41.4% to 49.7%. This follows the expected behavior of the ROC no-discrimination line, which implies that there is about 50% chance that a randomly selected sample of a target class (tumor) has a higher estimated probability of being predicted than a randomly selected sample from a non-target class (normal). For each data set, Table 4 shows the highest AUC in bold. MDR gives the highest AUC for the classifications of ALL-AML and Prostate, and four ALL subtypes: BCR-ABL, E2A-PBX1, MALL and TALL. SVM gives the highest AUCs for the rest. In the former group of classifications, SVM underperforms MDR with AUC differences ranging from 1% to 25.2%, whereas in the latter group, MDR underperforms SVM with AUC differences ranging from 0.4% to 7.9%. Specifically, SVM does poorly in BCR-ABL with an AUC 25.2% lower than the best AUC, obtained by MDR, whereas MDR has the largest AUC difference in subtype Hyp > 50 with an AUC 7.9% lower than that of SVM, the best performer. For the lymphoma cancer, SVM ties with Bayes for the best AUC, 97.9%, while MDR is competitive with an AUC of 93.6%. For classification of subtypes E2A-PBX1, MDR and C4.5 both have an AUC of 100%. Neither MDR nor SVM perform well on breast cancer, with AUCs of 63.7% and 68.1%, respectively. In terms of overall average AUCs, SVM and MDR outperform the other learners. MDR performs best on six of the eleven classification test cases and has an average AUC over all eleven cases of 91.01%. The overall average AUC obtained from SVM is 90.07%, and SVM has the best performance for five of the data sets. However, the difference between the average AUCs of MDR and SVM is not statistically significant.

**Table 4:** AUC Comparisons.

Data set	C4.5	Bayes	3NN	SVM	ZeroR	MDR
ALLAML	72.00	97.90	87.50	98.00	42.60	**99.02**
Lung	93.00	97.70	96.50	**98.00**	48.5	97.61
Prostate	79.00	59.50	87.10	91.00	47.90	**92.30**
Lymphoma	77.00	**97.90**	81.30	**97.90**	40.8	93.61
Breast	66.00	52.20	59.50	**68.10**	46.5	63.65
BCR-ABL	59.10	49.80	75.10	63.30	41.40	**88.48**
E2A-PBX1	**100.00**	53.70	99.00	98.10	46.10	**100.00**
Hyp	79.50	60.20	93.30	**93.40**	47.70	85.47
MALL	89.00	49.80	86.50	85.00	49.70	**89.41**
TALL	99.80	61.50	94.40	98.80	47.20	**100.00**
TEL-AML1	92.50	70.60	97.90	**99.20**	49.10	91.60

Average	82.45	68.25	87.10	90.07	46.14	**91.01**

Therefore, we can conclude that MDR is at least competitive to the other learners. In addition, recall that MDR provides the additional information of ranking predictions. We note that, unlike other learners, the AUC obtained from Weka for SVM is based on an ROC curve created using a single point (e.g., see Figure 1). Since SVM is a binary classifier that is not designed for classification ranking, this may seem to be a reasonable way to estimate AUC. However, this method may result in an optimistic AUC for SVM whenever SVM has high accuracy. Another observation is that MDR's performance may associate with the class distribution in the sample data. When the class distribution is extremely unbalanced, MDR does better than other learners. For example, in all subtype classifications of ALL, for which MDR outperformed other learners, the proportion of the target class is at most 15%. In particular, the proportions of the target class for subtypes BCR-ABL, E2A-PBX1, MALL and T-ALL are 0.04, 0.09, 0.06 and 0.15, respectively. To further compare the performance of MDR and SVM, we ran additional experiments (on a PC with a 3.2 GHz Pentium Processor and 3.6 GByte of RAM) with the TIS (Translation Initiation Sites) data obtained from [10,11]. Although these are vertebrate genomic sequences and not gene expression data, the TIS data set differs from all of the above expression data sets in that it has a much higher number of instances (13,375) than the number of attributes (927) with a class distribution ratio of about 25%. Our results show that while both SVM and MDR give the same AUC of 0.82, the training time of SVM is 1080.39 sec., which is significantly higher than MDR's training time of 84.26 sec. This shows that SVM is suitable for high dimensional data while MDR appears to be robust for data with high dimension and volume.

## Conclusion

This paper investigates the potential of MDR, a recently developed ranking algorithm, for obtaining effective and informative tumor classifications from gene expression data. MDR gives promising results with an average AUC of 91.01% and accuracy of 90.01% over 11 types and subtypes of cancers. The results show that MDR is competitive with other widely used high performance machine learners for microarray data analysis. Based on our experiments, MDR appears to be robust to unbalanced data and performs well even when the tumor class distribution is extremely unbalanced. In addition, MDR provides explicit assessment of classification rankings. MDR's accuracy in classifying different types of tumor is relatively high (close to 90%) on all types of cancer except breast cancer, where the accuracy and AUC obtained by MDR are about 63%. However, even SVM, which is the top performer for breast cancer, has accuracy and AUC of only about 68%. This implies that the breast cancer data used for the analysis may not have the structure and patterns that allow types of tumors to be discriminated. MDR performed very well on classifying all six subtypes of acute lymphoblastic leukemia, with the classification for subtype Hyp > 50 being the least accurate. Our work is preliminary and has been validated only on a limited number of data sets. Future work could include an investigation on the effects of the number of boosting rounds required by MDR during training, fine-tuning and experimentation with additional data sets to see how tolerant MDR is to noisy and inconsistent data. Further interpretation of the results obtained from MDR is required. Furthermore, since MDR can be viewed as a feature selection tool that is very simple and fast, it would be interesting to see if MDR can be applied for this purpose. These issues are part of our ongoing research.

## Methods

### The MDR algorithm

**Figure 2:**
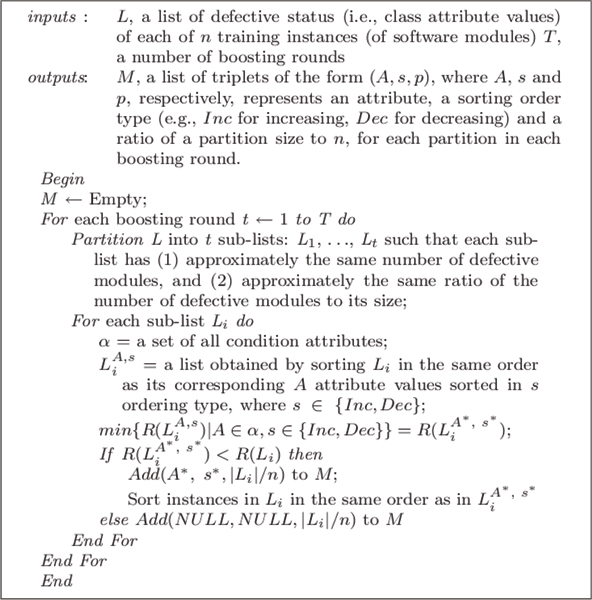
Basic steps of MDR

MDR (Multi-Dimensional Ranker) is a machine learning algorithm for constructing a predictive model (classifier) that ranks each sample in a data set based on its likelihood of being the member of a target class. In expression profiling data analysis, a typical target class represents a type of disease (e.g., a type or subtype of cancer). The algorithm is based on the Martingale boosting technique [14]. Intuitively, boosting iteratively uses results from one learner to tune the training instances so that learning in the next boosting round can incrementally improve prediction accuracy. Figure 2 shows basic steps of MDR.

In each boosting round, MDR performs a greedy search to select an appropriate attribute and its sorting order (increasing or decreasing) in each partition to improve the ranking pattern of target class samples in a given training data set. The selection is biased towards the ideal ranking, where samples are ordered from those with highest probability of being in the target class to the lowest. During the search, MDR uses a heuristic evaluation function, *R *as a rating function to compare lists (in a selected partition) sorted on different attributes in both increasing and decreasing orders. The partitioning condition ensures that the class attribute values (e.g., disease or non-disease) are well distributed in each partition for training. If the condition cannot be satisfied exactly, having the same number of target class instances is preferred (to having the same proportion of target class instances).

We now define the rating function for *L *= [*s*_1_, *s*_2_,..., *s*_*n*_], a list of samples in a partition of the training set. Let *d*_*i *_= 1 if a sample *s*_*i *_is a member of the target class and *d*_*i *_= 0 otherwise. We define rating of *L *as follows:

$$\begin{array}{ccc} R(L)=\sum_{i=1}^{n}r_{i}, & \text{where} & r_{i}=\{\begin{array}{ll} 0, & \text{if }d_{i}=1\\ \sum_{k=i+1}^{n}\frac{d_{k}}{n}, & \text{otherwise} \end{array} \end{array}$$

The rating is biased to ranking patterns that have target class members at the top of the list and non-target class members at the bottom. Ratings can range from 0 to *d*(*n *- *d*)/*n *where *d *is the total number of samples that belong to the target class. Note that smaller rating values are better. For example, consider lists *L*_1 _= [0, 0, 1, 1], *L*_2 _= [0, 1, 0, 1], and *L*_3 _= [1, 1, 0, 0]. It is clear that *L*_3 _is the most desirable since target class members are all towards the top of the list and *L*_1 _is the least desirable. This is consistent with the heuristic values: *R*(*L*_1_) = 1, *R*(*L*_2_) = 3/4, and *R*(*L*_3_) = 0.

**Figure 3:**
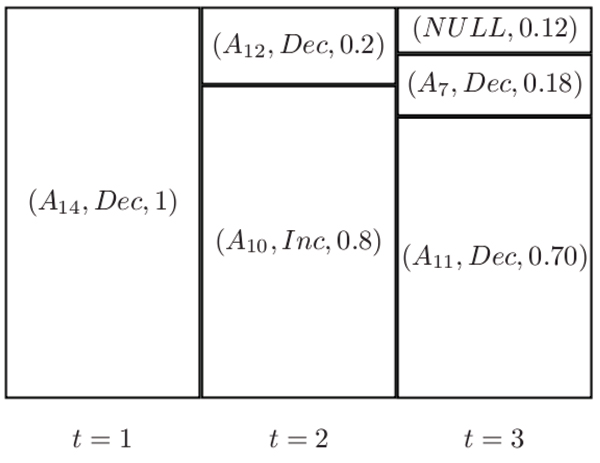
Example of a model produced by MDR

The output model is a list of triplets of the form (*A*, *s*, *p*), where *A*, *s *and *p *represent an attribute, a sorting order type (*Inc *for increasing, *Dec *for decreasing) and a ratio of the partition size to the number of training instances, respectively. Figure 3 shows an example of the model generated by MDR on a data set when the total number of boosting round is specified to be 3. The list of triplets for each partition in each boosting round is shown, where *A*_*i *_represents attribute *i *of the data set.

To apply the model to a given set of testing data instances, the list is repeatedly sorted according to the attributes selected by the model. For example, if we use the model in Figure 3, we will first sort the whole list according to attribute *A*_14 _in decreasing order. Next (in round 2), we partition the sample list into two parts with the top part containing 20% of the total number of testing samples. We sort the top partition by attribute *A*_12 _in decreasing order and sort the bottom partition by attribute *A*_10 _in increasing order.

Finally, in round 3, we partition the list into three parts: the top and middle parts contain 12% and 18% of the total number of testing instances, respectively. The rest are in the last partition. In the top partition, NULL signifies that no attribute is selected and therefore there is no sorting required. Thus, this top partition remains unchanged. We then sort the middle and bottom partitions in decreasing order by attributes *A*_7 _and *A*_11_, respectively. The resulting list gives the ranking predictions.

Unlike most machine learners, MDR is specifically designed to produce ranking models. The proposed MDR algorithm is similar to the MartiRank algorithm employed in the ROAM system [15] in that both are based on Martingale Boosting [14]. However, MDR differs slightly from MartiRank in that it uses a different heuristic evaluation function. Furthermore, MDR records each partition boundary in terms of the ratio of its size to the total number of training instances, whereas MartiRank uses an absolute boundary location. Consequently, MDR is more general than MartiRank in that MDR can be applied to testing data sets of any size whereas MartiRank can only be applied to those with the same size as the training data set. Currently, MDR, implemented in Java, interfaces, at certain levels, with Weka, a popular data mining tool [12] in order to facilitate valid comparisons of results obtained from MDR and other machine learners provided by Weka.

### The performance metrics

To evaluate the quality of our classification models, we employ various standard performance measures; ideally, different measures provide different insights. In machine learning, modeling involves classification or prediction that associates patterns from data points with classes that express different concepts. One of the most popular performance measures in machine learning classification is *accuracy *(ACC), which is defined to be the ratio of the number of correct predictions to the total number of instances in the test sample. However, ACC does not make use of the nature of the incorrect predictions, which can be useful in many domains including tumor classification.

Consider, for example, a model that classifies data points into the binary classes: *positive *and *negative*, as shown in Figure 2. *TP *(True Positive) represents the number of positive samples correctly classified, whereas *FP *(False Positive) represents the number of negative samples incorrectly classified as positive. *TN *(True Negative) and *FN *(False Negative) are defined symmetrically. ACC is computed as (*TP*+*TN*)/(*TP*+*FP*+*TN*+*FN*) and does not take into account the difference between *false alarms *(*type I errors*), measured by *FP*, and *missed detections *(*type II errors*), measured by *FN*. The latter value is particularly important in clinical medicine and defect management. Furthermore, ACC can be especially misleading when the distribution of the sample class is unbalanced. For example, a model can achieve 90% accuracy on a sample in which only 10% of the data points are positive simply by classifying every instance as negative, even though all of its classifications for positive samples are incorrect.

**Figure 4:**
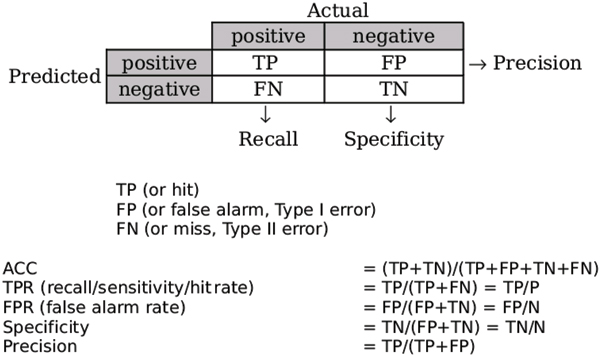
Performance metrics in binary classification

As shown in Figure 4, a variety of metrics have been defined to measure different types of errors. In particular, *TPR *(True Positive Rate) is defined to be the ratio of positives correctly classified to the actual number of positives, and *FPR *(False Positive Rate) is defined to be the ratio of negatives incorrectly classified to the actual number of negatives. TPR is also called the *hit rate*, *recall*, or *sensitivity*. FPR is also referred to as the *false alarm rate*. Similarly, *specificity *is defined as the ratio of correctly classified negatives to the actual number of negatives. TPR and specificity are independent, i.e., neither one tells us anything about the value of the other. Note that the sum of specificity and FPR is one, and thus, knowing one allows us to calculate the other. Another useful metric is *precision *(PREC), the ratio of correctly classified positives over the total number of samples classified as positive.

In this paper we also use *AUC *(Area Under the ROC Curve) as an additional performance metric. The ROC curve is a two-dimensional plot between the TPR (Y-axis) against the FPR (X-axis) of the predictions. The values of TPR and FPR range from zero to one. The set of points (FPR, TPR) defines the ROC space. Given a ranking list of sample predictions, each sample point in the list can be used as a cutoff point. For each cutoff point, the corresponding TPR and FPR can be computed and plotted as a single point in ROC space. Connecting these points gives the ROC curve for the list of predictions. ROC curves can be used to compare predictions. The closer the curve is to the Y-axis (high true positives) and the further away it is from the X-axis (low false positives), the more accurate the predictions are. When predictions are made by random guessing, TPR and FPR grow linearly yielding an ROC curve, which is a straight line 45 degrees from the horizontal. This is referred to as the no-discrimination line [13].

**Figure 5:**
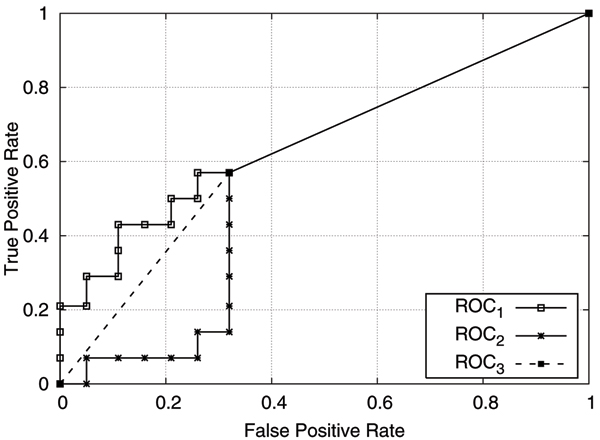
ROC curves on breast cancer classification of the same accuracy

Unfortunately, using ROC curves for performance comparisons does not necessary give conclusive results since one ROC curve may dominate another in one region but be dominated in a different region. According to [13], the AUC of a learner can be interpreted as the probability that, given a randomly chosen positive and negative example, the learner will give a higher ranking to the positive example than to the negative. Thus, AUC (the area under the ROC curve) is used as a value to compare the performance of different learners on a data set. The higher the AUC, the better the learner. Our study uses AUC to evaluate ranking quality [13]. AUC has been shown to be equivalent to the Wilcoxon statistic rank test [16] and, in fact, is a better measure for evaluating predictive ability of machine learners than accuracy [13]. Most researchers have now adopted AUC as the standard performance metric. Figure 5 illustrates three ROC curves obtained by applying the same classification model to the same testing sample using either different orderings for the testing data or using a different number of cutoff points. In particular, *ROC*_1 _and *ROC*_2 _are plotted using multiple cutoff points as the model incrementally classifies each testing instance, whereas *ROC*_3 _uses a single point to create the ROC curve with a cutoff point where all testing instances have been classified. *ROC*_1 _and *ROC*_2 _are obtained from the same testing instances but in different orders. Even though all the three classification results have the same accuracy, they have different AUCs. AUC is more sensitive to the order of classification ranking than it is to accuracy.

### Microarray data

We use microarray gene expression data for leukemia, lung, prostate, lymphoma, and breast cancers. These are real-world expression profiling data sets that are used in various types of cancer research [1,3,5-8,12]. They can be obtained from the Kent Ridge Biomedical Data Set Repository [11].

Table 5 gives a summary of the basic characteristics of these data sets, including the subject of the study, number of features (which represent, for example, genes or probing conditions of the microarray profiling experiments) and number of samples (cells or tissues from different patients). Each data set is concerned with a binary classification for different types or subtypes of cancers. The last two columns of Table 5 show the names of the target (e.g., tumor or type of cancer) and non-target classes and the number of instances of each. All data represent the expression levels and durations of the expressed genes. The last six rows of Table 5 represent expression data for binary classifications of six different subtypes of acute lymphoblastic leukemia (ALL). One characteristic shared by all these expression data sets is high dimensionality (many factors) but low volume (few sample points). As shown in Table 5, the dimensions range from 4,026 to 15,154, whereas the volumes range from 47 to 327. This characteristic is well known to challenge many existing microarray data analysis techniques.

**Table 5:** Information on cancer expression data.

Name	#Attributes	# Inst.	Target Class Name (# Inst.)		The other Class Name (# Inst.)	
ALL-AML Leukemia	15154	72	ALL	47	AML	25
Lung cancer	12533	181	MPM	31	ADCA	150
Prostate cancer	12600	136	Tumor	77	Normal	59
Lymphoma	4026	47	Germinal	24	Activated	23
Breast cancer	24481	97	Relapse	46	Non-relapse	51
ALL (Acute Lymphoblastic Leukemia)-subtypes	12558	327	BCR-ABL	15	non-BCR-ABL	312
	12558	327	E2A-PBX1	27	non-E2A-PBX1	300
	12558	327	Hyp	64	non-Hyp	263
	12558	327	MALL	20	non-MALL	307
	12558	327	T-ALL	43	non-T-ALL	284
	12558	327	TEL-AML1	79	non-TEL-AML1	248

### Experimentations

To avoid overfitting, *n-fold cross-validation *[12], a standard re-sampling technique, is used. In *n*-fold cross validation, a data set is randomly partitioned into *n *approximately equally sized subsets (or folds or tests). The learning algorithm is executed *n *times; each time it is trained on the data that is outside one of the subsets and the generated model (classifier) is tested on that subset. The estimated accuracy for each cross-validation test is a random variable that depends on the random partitioning of the data. The estimated accuracy is computed as the average accuracy over the n test sets. Typically *n*-fold cross-validations are repeated several times to assure data randomness, and the estimated accuracy is the average over these repetitions. In this paper, we use the number of folds, *n *= 10, as is standard practice. For efficiency control, MDR allows a user to specify the number of boosting rounds (*T*). In this study, we use *T *= 5 as the number of rounds. A method for selecting the best *T *is a subject for future research. We ran 10-fold cross validations on all eleven test cases of the six data sets using our MDR ranking algorithm and five other machine learning approaches, which are available on Weka [12]. The other five machine learners are *ZeroR*, *C4.5*, *Bayes*, *k-NN *and *SVM *[2,12,17,18]. ZeroR is a majority learner that is commonly used to provide a baseline measure of performance in machine learning. C4.5 is a decision tree learner, Bayes is a well known Naive Bayes classifier, and *k*-NN is a nearest-neighbor classifier, for which we specify the number of neighbors to be *k *= 3 (as in [9]). The support vector machine learner, SVM, is known to have high performance and is widely used in Bioinformatics. The five learners were selected for comparison because they perform well and cover a variety of techniques that use different representational models; for example decision tree models for C4.5, probabilistic models for Bayes, and regression models for SVM.

## Competing interests

The authors declare that they have no competing interests.

## Authors' contributions

RH designed the study, performed analysis of the micro array data and wrote the draft manuscript. PK implemented the proposed algorithm, performed the experiments and assisted in the analysis of the data. Both authors participated in production of the final version of the manuscript, read it and approved it.
